# Anti-Müllerian hormone plasma concentration in prepubertal ewe lambs as a predictor of their fertility at a young age

**DOI:** 10.1186/1746-6148-8-118

**Published:** 2012-07-23

**Authors:** Belén Lahoz, José L Alabart, Danielle Monniaux, Pascal Mermillod, José Folch

**Affiliations:** 1 Unidad de Tecnología en Producción Animal, Centro de Investigación y Tecnología Agroalimentaria (CITA) de Aragón, Zaragoza, 50059, Spain; 2 Physiologie de la Reproduction et des Comportements, UMR 085 INRA-UMR 6175 CNRS-Université de Tours-IFCE, Centre INRA de Tours, Nouzilly, 37380, France

## Abstract

**Background:**

In mammals, the ovarian follicular reserve is highly variable between individuals and impacts strongly on ovarian function and fertility. Nowadays, the best endocrine marker of this reserve in human, mouse and cattle is the anti-Müllerian hormone (AMH). The objectives of this work were to determine whether AMH could be detected in the plasma of prepubertal ewe lambs and to assess its relationship with their fertility at a young age.

**Results:**

Plasma was taken from 76 Rasa Aragonesa ewe lambs at 3.6 months of age for AMH determination. Simultaneously, 600 IU equine chorionic gonadotropin (eCG) was administered and the number of ovulations recorded 6 days later. AMH was detected in 93% of the lambs, and the concentrations were about 3–4-fold higher in ovulating than in non-ovulating lambs (*P* < 0.004). Ewes aged around 10 months were mated, giving an overall fertility of 29%, and those failing to conceive were mated again 4 months later. Fertility at first mating was significantly correlated with plasma AMH concentration at 3.6 months (Spearman’s ρ = 0.34; *P* < 0.01). To use plasma AMH concentration as a screening test, a value of 97 pg/mL was determined as the optimum cutoff value to predict fertility at first mating (sensitivity = 68.2%; specificity = 72.2%). Fertility at first mating was 34.8 percentage points higher in ewe lambs with an AMH ≥ 97 pg/mL than in those with lower AMH concentrations (50% vs. 15%; *P* < 0.001).

**Conclusions:**

Plasma AMH concentration might be a reliable marker of the ovarian status of prepubertal ewe lambs, reflecting their ability to respond to eCG stimulation. A single AMH measurement performed on ewe lambs early in age could be useful to select for replacement ewes with a higher predicted fertility at first mating.

## Background

The successful mating of ewe lambs within their first year of age reduces the effective cost of replacement ewes, increases the lifetime productivity of ewes in the flock, reduces the generation interval [1] and reduces the culling risks [2]. Moreover, an earlier first lambing brings about a higher productivity of the ewe throughout its life [3]. In some breeds such as the Rasa Aragonesa, even though females reach an adequate live weight at 12–13 months, the mean age at first lambing is 17–18 months [3], leading to extended unproductive periods. Therefore, it is challenging to find endocrine markers that could help to select the most precocious ewes, particularly if they could be used very early in life.

Sheep, as other mammals, are born with a limited and highly variable number of ovarian germ cells, which decreases drastically with age [4]. At birth, the oocytes are mostly contained in resting primordial and early growing follicles that constitute the so-called follicular reserve. Genetic factors and maternal nutrition during gestation both seem to contribute to this inherently high variation in the follicular reserve in sheep [4-8]. At 12–14 weeks after birth, an important population of gonadotropin-sensitive follicles is already present in the ovary [9,10]. Moreover, recent results suggest that the number of growing antral follicles present in the ovaries of young adult cattle may be related to their fertility [11]. Therefore, the follicular populations present in the ovaries of young ruminants seem to have a great impact on their adult reproductive life.

Presently, anti-Müllerian hormone (AMH) is the best endocrine marker of the ovarian follicular reserve of growing follicles, and it is used as a predictor of the ovarian response to gonadotropins in the human [12,13], mouse [14] and bovine species [15]. In the cow, AMH concentration in plasma is highly correlated with the number of antral follicles of 3 to 7 mm in diameter, which are the main targets of superovulatory treatments [15]. Also, AMH measurement can help to predict the aptitude of individuals to produce high or low numbers of embryos after superovulation, a repeatable and possibly heritable trait in the cow [16]. Recently, similar results were reported in mares, indicating that AMH could be a predictor of the number of follicles available in a follicle aspiration program [17]. Although a direct relationship between AMH and fertility has not been reported to date in domestic animals, some evidence supports this idea. On one hand, in young adult cattle, a high degree of correlation among AMH, antral follicle count (AFC) and the number of healthy follicles and oocytes in ovaries has been demonstrated; and on the other hand, lower numbers of antral growing follicles (AFC) have been related with suboptimal fertility [11]. Up to now, there are no data on plasma AMH concentration in sheep, though it would be of great interest for the future selection of animals for embryo production, as well as of young animals with a high fertility in the adult age.

Therefore, the aims of the present study were, first, to determine if AMH can be detected in the plasma of prepubertal Rasa Aragonesa ewes and to evaluate its relationship with the ovarian response to a gonadotropin treatment, and second, to relate AMH with fertility at first and second mating opportunities. With this aim, the plasma AMH concentration of ewe lambs aged around 3.6 months was assessed as well as the ovarian response to an equine chorionic gonadotropin (eCG) challenge, and their fertility was established after mating at 10 and 14 months.

## Results

### Plasma AMH concentration and its relationship with eCG-induced ovulation in prepubertal ewe lambs

AMH was detected in the plasma of 71 out of 76 ewe lambs (94%). The concentrations were highly variable between animals, ranging from 0 (*n* = 5) to 590 pg/mL. The age and weight of the ewe lambs with nondetectable AMH concentrations ranged from 88 to 126 days and from 23.5 to 28 kg. AMH was detected in the youngest lamb of this study (68 days) as well as in the lightest lamb (19 kg), reaching 362.5 and 53.7 pg/mL, respectively.

AMH concentration in plasma was not correlated with age (ρ = −0.19; NS) or live weight (ρ = −0.08; NS). Plasma AMH concentration was correlated with the occurrence of ovulation in response to eCG (ρ = 0.42; P < 0.0001) but not with the number of ovulations when only considering ovulating ewes (ρ = 0.08; NS). Neither the occurrence of ovulation nor the number of ovulations of ovulating ewes were correlated with age (ρ = 0.13 and −0.27, respectively; both NS) or live weight (ρ = 0.04 and 0.02; both NS). As expected, age and live weight were highly correlated (ρ = 0.38; P < 0.001).

Non-ovulating ewe lambs had lower plasma AMH concentrations than ovulating ewe lambs with 1, 2 or ≥ 3 ovulations (all P < 0.004; Table 1). However, no significant differences in AMH were found between the different groups of ovulating ewe lambs. No significant differences in live weight or age were found between any of the groups.

**Table 1:** **Plasma anti-Müllerian hormone (AMH) concentration (pg/mL), age (d) and live weight (kg) at the moment of ovarian stimulation with 600 IU eCG, for each ovulation group (0, 1, 2 and ≥3 ovulations**) **of prepubertal ewe lambs (means ± SEM)**

**No. of ovulations**	**No. of ewes**	**Age**	**Live weight**	**AMH**
0	19	108 ± 2 ^a^	25.0 ± 0.4 ^a^	43.0 ± 14.6 ^a^
1	17	118 ± 4 ^a^	25.5 ± 0.6 ^a^	126.9 ± 34.0 ^b^
2	21	104 ± 5 ^a^	24.7 ± 0.4 ^a^	99.8 ± 18.3 ^b^
≥3	19	108 ± 4 ^a^	24.7 ± 0.8 ^a^	163.1 ± 41.0 ^b^

Within columns, means with different superscripts differ significantly: a,b: P < 0.004.

### Relationship between plasma AMH concentration of prepubertal ewe lambs and their fertility at the first and second service periods

Ultrasound pregnancy diagnosis was coincident with fertility results both in the first (28.9%, n = 22 pregnant ewes) and in the second mating (77.8%, n = 42 pregnant ewes), at 10 and 14 months, respectively, revealing the absence of abortions and of mistakes in the lambing records. The overall fertility after the two consecutive mating opportunities was 84.2%.

**Figure 1:**
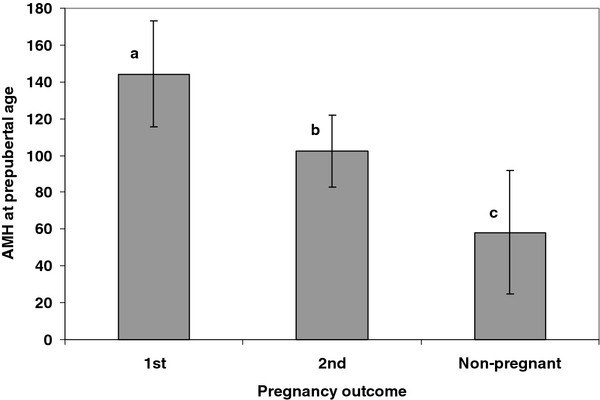
**Relationship between AMH at prepubertal age and pregnancy outcome.** AMH: Anti-Müllerian hormone plasma concentration (pg/mL) at prepubertal age (means ± SEM). Pregnancy outcome: 1st, ewes pregnant at first service (n = 22); 2nd, ewes pregnant at second service (n = 42); Non-pregnant, ewes failing to conceive after being mated twice four months apart (n = 12). Means with different superscripts differ: a,c: P < 0.01; a,b or b,c: P < 0.05.

The fertility of adult ewes at first, at second and after both consecutive service periods was correlated with their AMH concentration in the prepubertal phase (ρ = 0.34, P < 0.01; ρ = 0.33, P < 0.05; ρ = 0.36, P < 0.01, respectively). At the first mating, neither age nor live weight was significantly different between pregnant and non-pregnant ewes (315 ± 5 vs. 311 ± 3 days and 33.4 ± 0.6 vs. 32.6 ± 0.5 kg, respectively; both NS). Prepubertal plasma AMH concentration was higher in ewes that became pregnant at the first mating than in those which failed to conceive at first but became pregnant at the second mating (P < 0.05) or in non-pregnant ewes after two service periods (P < 0.01). It was also higher in ewes which became pregnant at the second mating than in non-pregnant ewes (P < 0.05; Figure 1).

**Figure 2:**
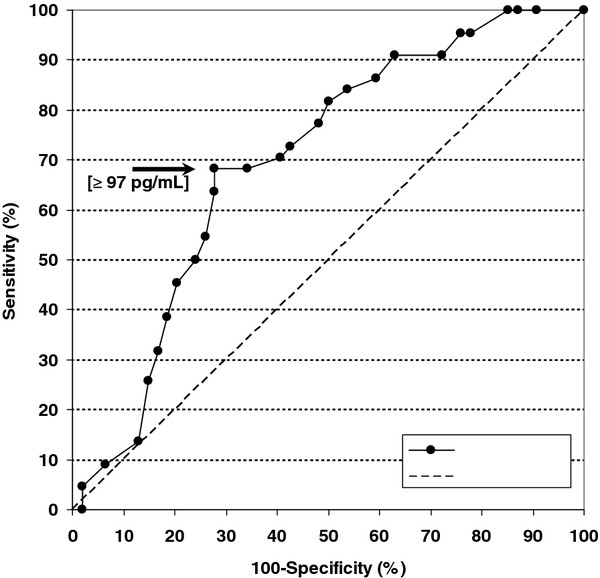
**Receiver-operating characteristic (ROC) curve for predicting fertility at first mating through AMH in prepubertal age.** The arrow shows the cutoff point maximizing the Youden index, and its corresponding AMH concentration is shown within brackets. A test without capacity of discrimination (random test) is represented as a dashed line.

The performance of AMH concentration at prepubertal age for predicting the fertility of adult ewes at first mating is shown in Figure 2. The receiver-operating characteristic (ROC) curve represents the relationship between the sensitivity of the diagnostic test based on AMH concentration at prepubertal age to predict the occurrence of pregnancy in adults (true-positive percentage) and 100 minus the specificity of the test to predict the failure of pregnancy (false-positive percentage) at first mating.

The predicting performance of plasma AMH concentration, assessed by the AUC (area under the ROC curve), was 0.698 ± 0.063, significantly different from 0.5 (the AUC value corresponding to a non-informative or “random test”) (P < 0.01). The AUC values estimated by the leave-one-out validation and 10-fold cross-validation methods were somewhat lower (0.660 ± 0.066 and 0.650 ± 0.013, respectively), although also different from 0.5 (P < 0.02 and P < 0.001, respectively).

The cutoff point of plasma AMH concentration that could be used for a screening test of the future ability of ewes to get pregnant at first mating was found to be 97 pg/mL. This AMH concentration corresponded to a sensitivity value to predict the occurrence of pregnancy of 68.2% (15/22) and a specificity to predict the failure of pregnancy of 72.2% (39/54). The positive and negative predictive values (PPV and NPV) were 50.0% and 84.4%, respectively (Table 2). The fertility at first mating of ewes with AMH concentrations equal or higher than 97 pg/mL before puberty was 34.8 percentage points higher than that of ewes with lower AMH concentrations (P < 0.001).

**Table 2:** Number and percentages (between brackets) of lambing and non-lambing ewes at first mating predicted through their plasma AMH concentration at prepubertal age using a cutoff value of 97 pg/mL

**Selection criterion**	**AMH group**	**Lambing**	**Non-lambing**	**No. of ewes**
AMH	≥ 97 pg/mL	15 (50.0) ^a^	15 (50.0)	30
	< 97 pg/mL	7 (15.2) ^b^	39 (84.8)	46
No selection	---	22 (28.9)	54 (71.1)	76

Within columns, percentages with different superscripts differ: a,b: P < 0.001.

The fertility at second mating or after both consecutive service periods was also higher in ewes with AMH concentration equal or higher than 97 pg/mL before puberty (93.3 vs. 71.8%; P < 0.03 and 96.7 vs. 76.1%; P < 0.004, respectively).

**Figure 3:**
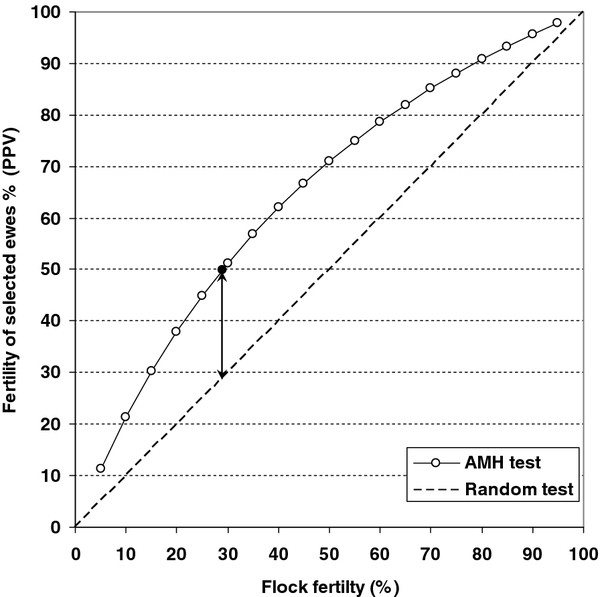
**Fertility of the selected ewes as a function of the flock fertility at first mating.** Data represent the predictive positive values (PPV, open circles). PPV values were calculated using the sensitivity and specificity corresponding to the cutoff point (plasma AMH ≥ 97 pg/mL; Se: 68.2%; Sp: 72.2%). The closed circle shows the PPV value corresponding to flock fertility at the first mating in the present study (28.9%). A test without capacity of discrimination (random test) is represented as a dashed line. The arrow shows the increase in fertility obtained in the present work by selecting ewes with AMH ≥ 97 pg/mL.

In the present work, the benefit of selecting replacement ewes by AMH (calculated as the difference between PPV, 50% and overall flock fertility at first mating, 28.9%), was 21.1 percentage points. As PPV (as well as NPV) depends on the prevalence of the character in the population [18] (herein, the overall flock fertility), PPV for assumed flock fertility values at first mating from 5 to 95% has been depicted in Figure 3. It is shown that the benefit obtained selecting replacement ewes by prepubertal AMH would be higher for intermediate than for either high or low flock fertility values.

## Discussion

This study shows for the first time that: (1) AMH is present in the plasma of prepubertal ewe lambs; (2) plasma AMH concentration is related to the occurrence of ovulation in ewe lambs after administration of an ovarian stimulation treatment, possibly reflecting that a population of gonadotropin-responsive follicles is already present at this age in most ewes; and (3) plasma AMH concentration before puberty could be used as a predictor of the fertility of adult ewes at first mating. This test would allow farmers to perform a precocious selection of replacement ewe lambs with the highest expected fertility at first mating. In the conditions of the present study, plasma AMH concentrations did not depend on age and weight, being detectable in most ewe lambs. Nevertheless, longitudinal studies would be necessary to accurately determine the age and weight at which AMH becomes detectable as well as the precise relationships of AMH with age and live weight.

In ewe lambs at around 3.6 months of age, plasma AMH concentration was about 3–4-fold higher in ovulating than in non-ovulating ewe lambs after eCG treatment. This difference could be due to the presence of a different numbers of antral gonadotropin-responsive follicles between animals at this age. In this way, the mean age of the ewe lambs of this study (15.6 weeks) would be coincident with a period where a peak in the total number of follicles ≥ 3 mm has been observed in other breeds of sheep, varying between 14 weeks [13] and 16 weeks [19]. Therefore, the relationship found between the ovulatory response to eCG and plasma AMH concentrations could be explained by the fact that they likely both reflect the presence of antral gonadotropin-responsive follicles in the ovaries of ewe lambs. In adult cows, a high correlation of plasma AMH concentrations with the number of corpora lutea after superovulation was found [15]. However, in the present study a relationship between AMH and the number of ovulations in ovulating ewe lambs was not found. This discrepancy could be attributed to physiological differences between prepubertal and adult females and/or to differences in the ovarian stimulation treatments. In this way, the ovarian response to a single eCG treatment without previous progestogen priming might be different to the response to a superovulation protocol consisting in multiple FSH decreasing doses with previous progestogen synchronization.

In the present work, neither plasma AMH concentration before puberty nor fertility at first mating was related with age or live weight. Moreover, all ewes received the same feeding plane during the whole experiment. Therefore, the observed differences in plasma AMH concentrations amongst ewes of similar age and similar live weight may be due to inherent variations in the follicular population, possibly influencing fertility at first mating. Genetic factors or maternal nutrition during pregnancy could therefore be responsible for this asynchronous follicular development; as in sheep, alterations in the maternal diet have been demonstrated to affect fetal ovarian development [5,7]. Nevertheless, further studies are needed to elucidate the causes of this asynchronous development of the ovary between the same aged and weighted ewes, as well as to determine whether it could affect ovarian function and reproductive performance during the adult life.

Apart from age, live weight and nutritional status, it is widely known that successful pregnancy is affected by many other factors, such as season, endocrine status or uterine conditions [20]. In the present work, healthy ewe lambs were reared under the same conditions to minimize the impact of such factors, although possible unknown individual disorders can not be discarded. The results of the present study showed that ewes with higher prepubertal plasma AMH concentrations displayed a higher probability of becoming pregnant at first mating than those with lower plasma AMH concentrations. Moreover, those ewes which failed to conceive after two consecutive mating opportunities had the lowest prepubertal plasma AMH concentrations. As far as we know, this is the first time that a relationship between AMH and early fertility has been established in a domestic species. Our study reinforces the prognostic value of AMH, even when the physiological status is not fully known. Both in the human and bovine, AMH levels are almost independent of the phase of the ovarian cycle, explaining why a single AMH measurement is usually sufficient [21,22]. This could be due to the fact that AMH is not involved in feed-back mechanisms of the hypothalamo-pituitary-gonadal axis [21]. Previous studies in the human have demonstrated a relationship between AMH and fertility. Very low concentrations of AMH are associated with infertility in the case of pre-menopause and premature ovarian failure, and very high AMH concentrations with an absence of ovulation in women with polycystic ovarian syndrome. Low or undetectable levels of AMH is explained by decrease or absence of follicular growth due to depletion of the ovarian reserve of primordial follicles. Abnormally high levels of AMH are explained by an excess of antral follicles whose growth is blocked prior to ovulation [21]. Recent results have also evidenced that AMH seems to be an excellent marker of ovarian function in girls and adolescents. In this way, elevated serum AMH levels have been found in girls with a precocious maturation of the adrenal cortex, suggesting that follicular development has proceeded to a more advanced developmental stage than expected for chronological age [23]. It has been demonstrated in adolescent patients with inherited reproductive problems (Turner syndrome) that their AMH concentrations correlate significantly with ovarian function at time of AMH measurement, showing an excellent sensitivity and specificity as a screening test of premature ovarian failure. Furthermore, it has been suggested that variations in AMH concentrations during childhood may theoretically predict the duration of any given girl’s reproductive lifespan [24], as AMH seems to reflect the continuous decline of the follicle pool with age [25].

In the case of ewe lambs, the observed AMH values would be rather a reflection of the beginning of follicular growth in prepubertal animals, showing an ovarian activity quantitatively different between animals of the same age. It is possible that ewe lambs in our study with the highest ovarian activity at 3.6 months were also the most precocious in terms of early puberty, resulting in the improved fertility observed when testing fertility of different ewes at the same age. The lambs with the earliest follicular development would be fertile at an early age. All these findings highlight the importance of individual differences in chronological follicular development and in the follicular reserve as being responsible for future reproductive life.

In sheep, recent in vitro studies have demonstrated that prepubertal oocytes isolated from ovaries with a high number of follicles larger than 2 mm are more competent for in vitro development, presenting a higher cleavage rate, developmental kinetics and cell number at the blastocyst stage [26]. As it has been established that the granulosa cells of large preantral and small antral healthy follicles produce the highest AMH amounts in rat [27] and cow [15,22], prepubertal ewe lambs with higher plasma AMH concentrations are supposed to have a higher number of healthy follicles in these stages, suggesting that the quality of the oocytes and embryos coming from these ewes may be higher. Although further studies are needed to confirm this hypothesis, it could partially explain the increased fertility at first mating of ewe lambs with the higher concentrations in plasma AMH during their prepubertal phase.

Concerning the ability of AMH to be used as a screening test to select the most precocious ewes in terms of fertility at first mating, in the present study the AUC value for AMH (0.698) was very close to 0.7, the limit value to classify tests as "moderately accurate" according to an arbitrary guideline. This guideline distinguishes between non-informative (AUC = 0.5), less accurate (0.5 < AUC < 0.7), moderately accurate (0.7 < AUC < 0.9), highly accurate (0.9 < AUC <1) and perfect tests (AUC = 1) [28]. The values obtained by cross-validation methods were somewhat lower and would correspond to a “less accurate” test. This is not strange as it its well known that the establishment and maintenance of pregnancy depends upon several genetic and environmental factors. Previous reports in the human have shown that while high AMH levels prior to the initiation of IVF treatments is a good predictor of the ovarian response, it has not proven to be as predictive of successful pregnancy. This indicates that factors other than the quantitative aspects of ovarian reserve may influence the fate of pregnancy [29]. Nevertheless, although not being highly accurate, we have shown that fertility of replacement ewes at first mating can be increased up to about 20 percentage points by using plasma AMH concentration as a screening test. It remains to be assessed at which age and weight the diagnostic test based on the measurement of AMH concentrations on ewe lambs is the most efficient for accurate prediction. Concerning fertility at the second mating and fertility after two consecutive service periods, we have shown that the differences between ewes with AMH concentration above and below the cutoff point are also significant and of great practical importance (+21.5 and +20.6 percentage points, respectively). This would indicate that AMH could also be a predictor of fertility in adulthood, but this needs further confirmation.

## Conclusions

In conclusion, a single AMH measurement in the plasma of prepubertal ewe lambs may be useful to select, in a very early age, the replacement ewes with a high predicted fertility at first mating.

## Methods

All experimental procedures were performed in accordance with the guidelines of the European Union (2003/65/CE) and Spanish regulations (RD 1201/2005, BOE 252/34367-91) for the use and care of animals in research.

### Experimental design

Prepubertal ewe lambs (aged around 3.6 months) were sampled for plasma AMH determination. In order to test the presence of gonadotropin-sensitive follicles at this age, an injection of eCG was applied, and 6 days after, the induced ovulation (number of ovulations) was recorded by laparoscopy. The relationship between AMH concentration and the ovarian response to eCG was investigated. Seven months later, the same young ewes were first joined to rams twice, 4 months apart. The relationship between fertility at these mating opportunities and plasma AMH concentration before puberty was studied. A ROC curve for AMH was analyzed to find the best cutoff value to predict fertility at first mating.

### Plasma sampling and determination of eCG-induced ovulation in prepubertal ewe lambs

The experiment started in July (beginning of the breeding season) in the facilities of the research center (CITA). A total of 76 Rasa Aragonesa prepubertal ewe lambs aged 109 ± 18 days (mean ± SD) and weighing of 25.0 ± 2.4 kg (mean ± SD) were used. Lamb ewes were weaned at 50 days postpartum and fed at libitum with concentrate until the beginning of the experiment. Ewe lambs received eCG (600 IU im; Sincropart PMSG, CEVA Salud Animal S.A., Barcelona, Spain), and simultaneously, blood samples were collected by jugular puncture using 5 mL vacuum tubes with lithium heparin that were immediately centrifuged at 2100 × g for 25 min. Plasma was stored at −20°C until analyzed for AMH. Six days after eCG treatment, ovulation was recorded by laparoscopy as follows. Ewe lambs were sedated with propofol (6 mL iv; Propofol Lipuro 1%, Braun, Spain) and local anesthesia with lidocaine was applied. An endoscope was inserted into the abdominal cavity through a 1-cm incision, approximately 10 cm cranial to the udder, and an atraumatic grasping forceps was introduced through a contralateral 1-cm incision. After total visualization of both ovaries, the number of healthy corpora lutea was recorded. The incisions were closed with staples and sprayed with topical chlortetracycline hydrochloride, and 1 mL/10 kg oxytetracyclinedihydrate (200 mg/mL; Oxycen-200 L.A., s.p.® veterinaria, s.a., Tarragona, Spain) was administered intramuscularly. After that, animals were fed at libitum under grazing conditions.

### Evaluation of the fertility of the ewes at first and second mating

In this study, fertility was defined as the ability of a ewe to become pregnant in a given mating period. In January (end of the breeding season), when ewes were 312 ± 18 days old (mean ± SD) and weighing 33.1 ± 3.1 kg (mean ± SD), they were first joined to four adult Rasa Aragonesa rams of proven fertility. The minimum ram-ewe lamb ratio was around 5% (first mating: 4/76; second mating: 4/54). This ratio was similar or slightly greater than those proposed by other authors [30,31]. Males were removed after 34 days. Fertility at first mating was calculated as the percentage of ewes lambing (and thus successfully mated) over ewes exposed to rams (n = 76) in this first service period. Four months later (in May), the non-pregnant ewes were mated again for 34 days, according to an accelerated lambing program of three lambings in 2 years. Fertility at second mating was calculated as the percentage of ewes lambing (and thus successfully mated) over ewes exposed to rams (not considering pregnant ewes at first mating) in this second service period. Overall fertility after the two consecutive mating periods was calculated as the percentage of ewes lambing after either first or second mating periods, over all the ewes exposed to rams (n = 76). Ten and 30 days after the rams’ removal, ultrasound diagnosis of pregnancy was performed. Lambing took place in individual pens and offspring were immediately identified.

### AMH assay

**Figure 4:**
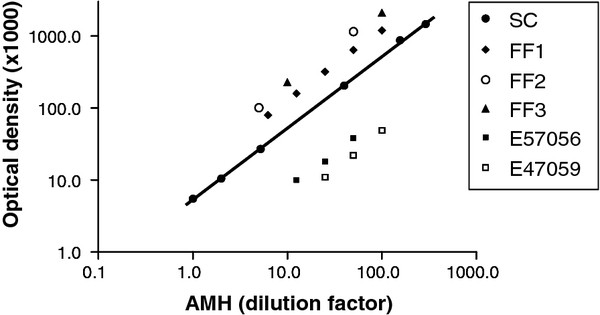
**Parallelism of dilution curves of ovine plasma and follicular fluids with the human AMH standard curve in the Active MIS/AMH ELISA kit.** The figure depicts the results of the serial dilutions of ovine follicular fluid samples (FF1, FF2 and FF3), plasma from 2 ewes (E57056 and E47059) and the standard curve (SC) of the AMH ELISA. Dilutions of the standard points and the ovine samples were all made in steer plasma. The absorbance (optical density) measured at 450 nm at the end of the test is directly proportional to the concentration of AMH in the samples.

Plasma concentrations of AMH were measured with the Active MIS/AMH ELISA Kit (Beckman Coulter France, Roissy CDG, France), as described previously [15,32]. Just before the assay, the frozen plasma samples were thawed in a warm water bath, vortexed and centrifuged (3200 g, 10 min, 4°C) to remove any cell fragments that could interfere with the reagents of the assay. AMH concentrations were determined in 50-μL undiluted plasma samples. The samples were incubated overnight at 4°C in the presence of the primary antibody, then for 1.5 h at room temperature in the presence of the secondary antibody. Dilutions of the points of the standard curve were made in steer plasma. With these conditions, the limit of detection of the assay was found to be 15 pg/mL. All the plasma samples were analyzed in the same assay. The intra-assay coefficients of variation were all lower than 5 % for the four quality control plasma samples that were tested, containing 27, 112, 195 and 274 pg/mL of AMH. For further validation of the assay, serial dilutions of different ovine plasma and follicular fluid samples in steer plasma were analyzed. Results showed that dilution curves were linear and parallel to the standard curve (Figure 4).

### Evaluation of plasma AMH concentration in prepubertal age as a predictor of fertility of the ewe at first and second mating

An ROC analysis was performed to evaluate the predicting ability of plasma AMH concentration to detect the ewes with a high fertility at first mating. The sensitivity was defined as the ratio between the ewes correctly predicted as pregnant above an AMH value and all the pregnant ewes. The specificity was the ratio between the ewes correctly predicted as non-pregnant below an AMH value and all the non-pregnant ewes. The PPV was the ratio between the ewes correctly classified as pregnant and those predicted as pregnant (in other words, the fertility of the selected ewes above an AMH value). The NPV was the ratio of ewes correctly classified as non-pregnant and those predicted as non-pregnant (100 minus fertility of non-selected ewes, below an AMH value).

### Statistical analysis

Correlations among the variables: occurrence of ovulation (1 = ovulating; 0 = non-ovulating), number of ovulations in ovulating ewes, plasma AMH concentration, age, live weight and fertility (1 = pregnant; 0 = non-pregnant) were assessed by the Spearman correlation coefficient (ρ). Continuous variables (age, live weight, plasma AMH concentration) were tested by general linear models (ANOVA). Normality of residuals was tested by the Jarque-Bera test, applying a correction for finite samples [33]. Homogeneity of within-groups variances (homoscedasticity) was tested by the Brown-Forsythe test. As plasma AMH concentration failed to accomplish the requirements of residuals normality and homoscedasticity, its values (expressed in pg/mL) were transformed to decimal logarithms (log_10_[AMH + 1]) prior to ANOVA tests. Pairwise comparisons between means were carried out by the least significant difference test, applying the “false discovery rate” approach [34] to adjust probabilities for multiple comparisons. For plasma AMH concentrations, although significance shown in tables was obtained through the transformed data, arithmetic means and SEM of the original, non-transformed data are presented. Dichotomous variables, presented as percentages in text and tables, were analyzed by chi-square tests. When the number of ovulations was used as independent variable in ANOVA, ewes with numbers of ovulations higher than two (from 3 to 7) were grouped into one category owing to the small number of ewes in these categories. In order to evaluate plasma AMH concentration as a predictor test of the fertility at first mating, the ROC curve was analyzed. The AUC was used as an indicator of the global performance of the test. The values of AUC were compared with 0.5 (AUC for a “random test”), using the non-parametric approach for testing statistical significance [28]. As AUC values computed from the data set used for model fitting tend to indicate better accuracy than the actual model allows in practice, AUC values estimated by the leave-one-out validation method and by 10-fold cross-validation method are also presented [35]. The 10-fold cross-validation was repeated 10 times and the average AUC of these 10 replicates ± SD was calculated. The cutoff value of AMH to discern between fertile and non-fertile ewes at first mating was chosen as the corresponding point of the ROC curve that maximized the Youden index, a prevalence-independent criterion [28].

All statistical analyses were performed using SAS software [36]. Otherwise specified, all data presented in text are means ± standard errors.

## Authors’ contributions

BL, JLA, PM and JF designed research. BL and JF administered treatments, performed endoscopy and collected samples and data. DM and PM analyzed samples. JLA carried out statistical analyses. BL, JLA, DM and JF have written the manuscript. All authors have contributed to the discussion. All authors read and approved the final manuscript.
